# Transgene flow in Mexican maize revisited: Socio‐biological analysis across two contrasting farmer communities and seed management systems

**DOI:** 10.1002/ece3.3415

**Published:** 2017-10-11

**Authors:** Sarah Agapito‐Tenfen, Flor R. Lopez, Narmeen Mallah, Gretta Abou‐Slemayne, Miluse Trtikova, Rubens O. Nodari, Fern Wickson

**Affiliations:** ^1^ GenØk Center for Biosafety Siva Innovasjonssenter Tromsø Norway; ^2^ Department of Laboratory Science and Technology American University of Science and Technology Ashrafieh Lebanon; ^3^ Institut für Integrative Biologie ETH Zurich Zürich Switzerland; ^4^ Departamento de Fitotecnia Universidade Federal de Santa Catarina Florianópolis Brazil

**Keywords:** biotechnology, genetically modified organisms, socio‐biological analysis, transgene flow

## Abstract

The flow of transgenes into landraces and wild relatives is an important biosafety concern. The case of transgene flow into local maize varieties in Mexico (the center of origin of maize) has been intensively debated over the past 15 years, including legal, political, and environmental disputes fanned by the existence of a significant scientific controversy over the methods used for the detection of transgenes. The use of diverse approaches and a lack of harmonized methods specific to the detection and monitoring of transgenes in landraces have generated both positive and negative results regarding contamination of Mexican maize with genetically modified material over the years. In this paper, we revisit the case of transgene contamination in Mexican maize and present a novel research approach based on socio‐biological analysis of contrasting communities and seed management systems. Two communities were used to investigate how different social and biological factors can affect transgene flow and impact transgene spread in Mexico. Our results show the presence of transgenes in one community and thus support the position that transgenes are highly likely to be present in Mexican maize landraces. However, our work also demonstrates that the extent and frequency with which transgenes can be found will significantly depend on the societal characteristics and seed management systems of the local communities. Therefore, we argue that future analysis of transgene presence should include social research on the seed management practices in the sampling area so that more robust and comprehensive understandings and conclusions can be drawn.

## 1 INTRODUCTION

Gene flow is an important evolutionary force that became a cross‐cutting issue of importance after the release of transgenic genetically modified (GM) organisms into open ecological systems (Ellstrand, 2003). The flow of GM transgenes and their introgression into the valuable germplasm of important crop plants and their wild relatives can cause unexpected consequences, impact conservation, and affect regulatory policies and crop management systems (Ellstrand, 2014). Indeed, transgene flow from GM crops into landraces and wild relatives is an issue that has rarely been out of the news since it was first claimed that transgenes had been detected in Mexican maize in late 2001 (Quist & Chapela, 2001). After this initial study became the subject of significant scientific debate and extensive socio‐political controversy, several others have attempted to answer the question of whether transgenes could be detected in maize at its center of origin (despite there being a moratorium on growing GM maize in Mexico) (e.g., Dyer et al., 2009; Ortiz‐García et al., 2005a, 2005b; Piñeyro‐Nelson et al., 2009a, 2009b; Serratos‐Hernández et al., 2007).

However, each study examining this issue has come to different conclusions, and the case has been fraught with disputes over what is the best or most appropriate and reliable detection method. Although the large‐scale commercial release of transgenic crops continues worldwide, there is still no scientific agreement on this iconic case of transgene flow into landraces of Mexican maize and a legal dispute is now ongoing over GM maize approval and production in Mexico (Garcia, 2017; Vargas‐Parada, 2014).

Several maize seed management systems have been described for various regions across Mexico, with different rates of seed replacement, introduction, and diffusion occurring across them (Badstue et al., 2006; Louette & Smale, 1998; Smale, Aguirre, Bellon, Mendoza, & Manuel Rosas, 1998). In addition, it has been suggested that the effect of farming practices on maize diversity and conservation can be influenced by geographic and other environmental factors, frequently defined in terms of latitude and altitude (Brush & Perales, 2007). However, there is currently limited research available that considers how social norms, values, and practices affect maize diversity and, consequently, its conservation (Brush & Perales, 2007). It is only recently that studies have begun to address the specific impacts of seed type, source, geographic region and ownership, and other human influences in seed sharing rates (Dyer & López‐Feldman, 2013). As GM transgenic maize seeds are genetically distinct from other types of seeds, as well as are produced in different geographic regions and have different ownership regimes to those seeds traditionally grown and commercialized in Mexico (Lacey, 2000), it is important to specify how social and biological factors may influence transgene flow in maize in Mexico.

Due to the different ownership regimes in play and the potential impacts of transgene flow on agricultural biodiversity conservation, it is crucial to develop research approaches that can help anticipate where, how, and why transgenes might be present in Mexican maize landraces and where spread is most likely to occur or to occur at a rapid rate, using not only knowledge of biological and geographical features but also knowledge of social practices and management systems (Dyer et al., 2009; Piñeyro‐Nelson et al., 2009a). Previous research groups have pointed out that future studies should therefore analyze the effect of contrasting production and seed management conditions on transgene frequency distributions and their detection probabilities (Piñeyro‐Nelson et al., 2009a).

However, screening maize within Mexico for transgene flow is a daunting task that poses many challenges. Transgenes can flow through both formal and informal seed systems and grain markets, as well as via the interactions between these channels, which can be particularly hard to track (Dyer et al., 2009). Therefore, we considered it relevant and timely to investigate how contrasting seed management systems could influence the potential presence and spread of transgenes in indigenous communities, using a mixed method approach that combines biological detection work with social surveys and interviews. Our analysis of the social and biological data combined highlights how the potential for transgenes to enter, survive, and disperse through informal seed systems varies significantly between the two communities under investigation. The main reason for this difference is related to their contrasting social norms and structures, such as those governing seed exchanges outside the community, the sourcing of seeds from local stores and markets, and the choice of variety for cultivation. Although we did not find transgene presence in landrace seed stocks in the year of sampling in one of the two communities we collaborated with, transgenes were revealed as present in the seed being sold in local markets and which our social analysis confirmed that farmers in the other community typically buy as grain and also sometimes plant and share as seed. Our research therefore demonstrates not only the real potential for transgene flow into indigenous communities and the landraces they typically cultivate in Mexico but also that this potential varies significantly with socio‐cultural norms and the structures and practices of these communities. Researching social‐biological interactions is therefore crucial for understanding the controversial question of transgene flow in Mexican maize.

## 2 MATERIALS AND METHODS

In order to understand the influence of seed management systems on the presence and flow of transgenes in maize in Mexico, this study employed a mixed method approach combining both natural and social science research methods across two different indigenous communities in the region of Oaxaca. This included conducting a social survey of volunteer farmers, supplemented by semistructured interviews and attendance at communitarian meetings, as well as environmental sampling and detection testing of the seed lots from the same volunteer farmers surveyed and interviewed, as well as from the local markets and stores where farmers purchase seed and/or grain. Each of these elements of the research method is outlined in more detail below.

### 2.1 Farmer communities

Two contrasting communities were selected for this study, both representing indigenous communities growing maize in Oaxaca (a center of maize origin and the location in which transgene presence was controversially first reported in Mexico (Quist & Chapela, 2001)). The communities differ significantly across a range of factors, including (1) seed saving and sharing practices, (2) community organization (e.g., the extent to which farming decisions are made by the community or by individuals), (3) land tenure arrangements, (4) proximity to urban developments, and (5) ethnicity. An overview of their approximate location^1^ and selected geographic and social characteristics are presented in Table 1.

**Table 1:** Contrasting characteristics of Communities A and B

Contrasting characteristics	Community A	Community B
Location	Valles Centrales de Oaxaca Region	Sierra Mixe, Istmo Region
Total population (at time of study)	3,616	981
Gender distribution	Women: 2,010, Men: 1,606	Women: 500, Men: 481
Number of occupied dwellings	896	218
Seed saving and sharing practices	Farmers grow landrace maize and hybrid maize. Farmers save seed and share outside the community	Farmers grow only landrace maize. Farmers save seed and share only inside the community
Communitarian organization	Farming decisions are taken at an individual level	Farming decisions are taken at a communitarian level
Land tenure arrangements	Fields are owned as individual property	Fields are communitarian property
Proximity to urban development	Close (36 km to the nearest city)	Distant (204 km to the nearest city)
Ethnicity	Zapoteco	Mixe

“Community A” is located relatively close to Oaxaca de Juárez, the capital of Oaxaca State and also to the neighboring municipality Ocotlán de Morelos. It is a Zapoteco indigenous community and although people still speak the Zapoteco language and maintain some of their indigenous traditions, the proximity to a major city center and temporal emigration to the United States have facilitated many cultural changes. Maize agriculture is still performed by small‐scale farmers who own their own plots and make decisions at the level of the individual farmer. In this community, hybrid seeds can be planted by any farmer without special notice needing to be given to neighboring farmers. In addition, maize plots of different farmers within the community are located side‐by‐side and can also border plots from farmers from other communities.

“Community B” is a Mixe community in the Sierra Mixe region. Mixe people have a strong history of organization, union and defense of their rights, territories, and autonomy (Blanco, 2012). In this community, the indigenous language remains widely spoken (especially among the older generations), and the community has a significant degree of autonomy. For instance, the community has their own police force, which is officially recognized by the Mexican government and is the only police force operating in the community. The community is geographically isolated from any other neighboring community and is approximately 75 km from the nearest municipality, Matias Romero Avendano. However, as in the rest of Oaxaca State, temporary emigration is frequent among young people. There is a communitarian management of the land, in which decisions on farming and land use must be taken through a general assembly. This means that maize plots are not privately owned and it is a communitarian assembly that decides who will farm which plot each year. The field plots are also isolated from neighboring plots by dense humid forest and mountains. Decisions made by the community over the past years have lead to a ban on the replacement of traditional seeds by hybrid maize or maize from outside the community.

### 2.2 Social survey and interviews

The research began by a presentation of the project at a communitarian meeting in each community and a call for volunteers. Those maize farmers volunteering were asked to complete a social survey as a way to collect an information profile on each of the volunteers. This included demographic questions on age and gender but also questions related to factors such as the size and geographic location of their maize plots and the history and source of their seeds. On the basis of this survey, a representative sample of 20 farmers from each community was selected for further social research and biological sampling of their maize seed stock. The sample of participants was selected from the pool of volunteers on the basis of the following criteria: (1) The farmer has sufficient maize stocks to donate a sample for testing (500 g to 1 kg or at least eight cobs), (2) the farmer has criolla (landrace) and/or acriolladas (OPV/landrace) maize seeds, (3) the farmer is part of the community (this facilitated feedback at the community level), and (4) the participants as a whole have a representative geographical spread across the community. All selected farmers relied on traditional seed systems and planted mostly maize landraces, although some of them had experimented with improved and hybrid varieties as well. All of the participants were asked to read and sign a consent letter for their involvement in the project, and the project was registered with the Data Protection Authority responsible for processing personal data in the country of origin of the project. Local authorities were also consulted about the project and both community authorities signed a consent letter approving the research in their community.

Follow‐up semistructured interviews were then conducted with the selected volunteer farmers to more deeply investigate their seed management practices and their knowledge and perceptions concerning GM maize. The researchers conducting the interviews were fluent in English and Spanish but also had the help of a community member fluent in Spanish and the indigenous language. Interviews were conducted by the lead investigator in Spanish or translated to Zapoteco or Mixe when necessary. The interviews were recorded, transcribed, and translated to English. These transcripts were then coded and analyzed using the program for quantitative and qualitative analysis known as Dedoose (http://www.dedoose.com/).

### 2.3 Environmental sampling

The objective of the environmental sampling was to determine the presence and frequency of transgenes in the landrace populations of maize in each community. This meant that it was necessary to use a sampling methodology maximizing the probability of finding rare alleles in the reference population and representative of that population. Although there is no validated standard method for sampling landraces for the purposes of transgene monitoring, given our aim to maximize the probability of finding rare alleles, we used the method proposed by Cleveland, Soleri, Cuevas, Crossa, and Gepts (2005), in which an equal number of seeds were sampled from each sampling unit (the farmer), and an equal number of seeds from the largest possible number of maternal plants were taken. Population representativeness was dependent on the choice of the volunteer farmers and therefore not completely random and spread across fields, seed type, etc. We used the formula for variance effective population size (Ne) according to Cleveland et al. (2005) and have estimated an Ne(v) of 596 seeds or at least 148.5 plants per community (in our case 7.4 cobs per farmer across a total of 20 farmers) necessary for detecting transgenes in a population in a hemizygous condition at a frequency of 0.01. Therefore, each sample consisted of a seed lot sample of 500 g in Community A and eight cobs in Community B, which was the equivalent of approximately 500 g (the difference being due to the variance in seed storage practices in each community—maize cobs are harvested and then degrained and stored in containers for the next season in Community A, while in Community B grains are stored on the cob until the next season). For Community A, an increased number of maternal plants were sampled due to the sampling of grains and not cobs; thus, we have increased the probability of finding rare alleles. As some of our farmers informed us during the interviews that they sometimes bought seed at neighboring markets outside their community or grain at the local stores (e.g., DICONSA), which they sometimes planted as seed, we also decided to sample these sources of seed flow into the communities.

### 2.4 Interlaboratorial analysis

Three laboratories were involved in the transgene detection analysis, one in Norway, one in Switzerland, and a third ISO 17025 accredited GMO testing laboratory in Lebanon. The three laboratories followed the same protocols for sample handling, subsampling, DNA extraction, and qPCR analysis. Samples were roughly ground in Mexico in an industrial blender (International model LE‐3) followed by cleaning and decontaminating procedures for each sample (ENGL, 2014) and further shipped to Norway. This process was necessary so that the material for analysis was not shipped abroad in a cultivatable form, which would be in violation of Mexico's biodiversity law. The roughly ground 500 g samples were then split into five subsamples of 100 g. Two subsamples were kept in 10°C for backup storage, and three subsamples were finely ground using an analytical grinder (IKA model A11). Three subsamples of 1.5 g were taken from each of the 100 g samples and shipped to each laboratory for testing. Although the 1.5 g aliquots represent different flour samples, homogenates of 1.5 g derived from this analytical grinder are representative of the 100 g sample. While all methods of sampling have associated limitations, the approach to subsampling chosen in this case was necessitated by the practical constraints of what was permitted under the Mexican biodiversity law, the equipment available onsite in Oaxaca, and what number of samples it was possible to analyze across all three laboratories with the resources available.

### 2.5 DNA extraction

The DNA isolation protocol was based on the CTAB method and followed ISO21571 guideline. An environmental negative control sample was used as well as an extraction blank control in between every 10 extractions to track potential cross‐contamination. Quality control measurements followed that of The European Network of GMO Laboratories (ENGL, 2015). An inhibition test was also performed according to ENGL guidelines (2011) to check for DNA purity and quality.

### 2.6 Real‐time PCR analysis

Qualitative transgene detection analysis was performed using real‐time PCR with protocols validated by the Joint Research Center (http://gmo-crl.jrc.ec.europa.eu/gmomethods/). Two commonly found transgenic elements were selected for this analysis, the Cauliflower Mosaic Virus 35S promoter (CaMV P‐35S; protocol reference QT‐ELE‐00‐001) and the Nopaline synthase terminator (T‐nos; protocol reference QL‐ELE‐00‐011) from *Agrobacterium tumefaciens*. A third element was also tested for internal control, the alcohol dehydrogenase1 gene (adh1; protocol reference QT‐TA X‐ZM‐001). The reference materials ERM‐BF415b and ERM‐BF415d were used as positive and reference controls. Cycle threshold (Ct) levels for each primer, as well as primer efficiency, were determined by standard curves, and baseline levels were set automatically for each piece of equipment. Relative limit of detection values (LOD_rel_ scores) was obtained below or equal to 0.1% using the reference material NK603 0.1% in each laboratory and followed the procedure of ENGL (2011). Finally, ten technical replicates derived from the same DNA extraction were re‐analyzed in cases where there was inconsistency in the results across the laboratories. Results of the ten replicates were scored “likely positive” when five or more samples presented cycle of quantification (Cq) values above threshold levels and “likely negative” when more than five Cq results were scored “undetermined” for each laboratory analysis.

## 3 RESULTS

### 3.1 Seed management systems

**Figure 1:**
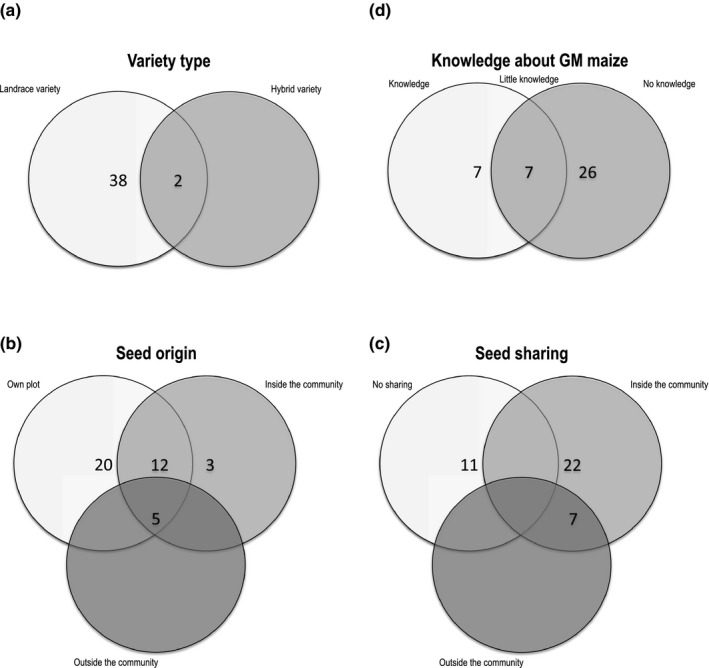
Data on (a) variety type, (b) seed origin, (c) seed sharing practice, and (d) knowledge about genetically modified (GM) maize information collected at Community A and B in Valles Centralles de Oaxaca and Istmo region in Mexico in 2015. *N* = 40

As we selected 20 farmers per community and our research was based on a volunteer basis, it was not possible to reach both a gender and an age balance in the group. For Community A, 35% were male farmers, 65% female, and the average age of participants was 43.5 years. For Community B, it was the opposite, 35% were female farmers, 65% male, and the average age of participants was 49.4 years (see Fig. 1a). Although three farmers from Community A stated that they grow both landrace and hybrid varieties, our research did not survey volunteers who exclusively grow hybrid or open pollinated varieties.

A high proportion of farmers across both communities (92.5%) recycle or save their own seed for the next season (Fig. 1b). However, 5% of farmers and 10% of farmers do not save their seeds in Community A and B, respectively. This was primarily due to their small plot size, which were sometimes smaller than average and then did not produce enough harvest for food and seed for next season's cultivation. Other farmers combined seed from different sources, for example, they supplemented their saved seed with seed bought or shared from inside the community (50% in Community A and 35% in Community B) or outside the community (10% in Community A and 15% in Community B). Sharing of seeds (giving away or selling) was found to be very common, and 72.5% of all the farmers reported to have frequently participated in this activity during their farming history (Fig. 1c). There was a higher proportion of farmers who did not share their seeds (40%) in Community B in contrast to Community A (15%). In Community A, farmers also reported frequently sharing their seeds outside of the community (30%) in contrast to only one farmer in Community B. Interestingly, some farmers in Community A reported buying grains from local stores (such as DICONSA) and growing them when no landrace seeds were left from their previous harvest. Retailers confirmed that some farmers in the region also sow maize grains that are not sold as “seed” but rather as food or feed.

Importantly, the majority of interviewed farmers (82.5%) did not know about GM maize or reported having just heard of it (Fig. 1d). This lack of knowledge did not differ significantly between the communities, even though Community A is only 35 km from the city of Oaxaca de Juarez, where much NGO work and popular communication around GMOs has taken place in previous years.

**Figure 2:**
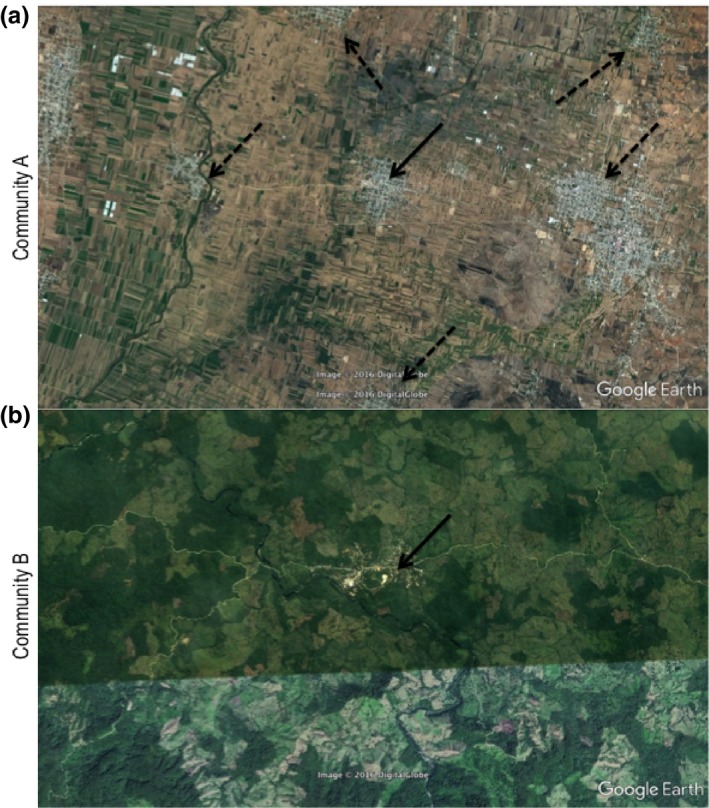
Geographic and size distribution of agricultural fields in Community A (a) and Community B (b). Full arrows indicate the community city center and dashed arrows indicate neighboring communities. Image taken from: Google earth V 7.1.8.3036 (7 March 2017). Data SIO, NOAA, U.S. Navy, NGA, GEBCO. http://www.earth.google.com (7 March 2017). Full geographic coordinates were deleted to preserve the anonymity of the communities' exact location. Scale: 1 cm represents approximately 1,000 m in Figure 2a and 1,800 m in Figure 2b

In Community A, the majority of farmers owned more than one plot, and plots were typically distributed across the community and measured in number of rows (*surcos*), whereas in Community B the farmers sowed in single but rather larger plots (measured in hectares). The size and geographic location of farmer's plots revealed the organization and density of maize fields of the interviewed farmers, which were distinct in each community (further confirmed by Google Earth imaging (Fig. 2a and b). It is important to note that the distinct distribution pattern may have implications for the spread of transgenic pollen across fields. In addition, in Community A neighboring communities demonstrate the same agricultural field pattern, and the borders between the plots of the different communities are not delimited by any geographic barriers.

### 3.2 Transgene detection

Transgene presence was investigated by searching for the two most common transgenic elements present in the GM maize varieties cultivated worldwide: the Cauliflower Mosaic Virus 35S Promoter (CaMV P‐35S) and the Nopaline Synthase Terminator (T‐nos). We applied certified methods for screening the two elements based on real‐time PCR, which are provided by the Joint Research Center and publicly available (http://gmo-crl.jrc.ec.europa.eu/gmomethods/). An interlaboratorial approach was undertaken by three independent laboratories to replicate the testing.

We tested seed lots from 40 farmers and 13 samples from local stores and markets. In total, 19 samples showed amplification for at least one of the transgenic targets in at least one laboratory. Screening results showed amplification for P‐35S target in 12 samples and in 10 samples for T‐nos target across laboratories (Table 2). Five samples (sample ID 15, 23, 27, 32, and 45) showed amplification of one target in at least two laboratories, and samples 15 and 32 were the only ones to show amplification in all three laboratories for the P‐35S target. There were also several cases in which only one repetition within a laboratory showed amplification (sample ID 2, 3, 8, 9, 10, 25, 26, 30, 42, 45, 47, and 55).

**Table 2:** Normalized cycle of quantification values (Cq) obtained for each sample by each of the three laboratories involved in this study. The results for the two transgenic targets (P35S and TNOS) are presented in separate columns. “Undetermined” results were obtained when no fluorescent signal was detected by the real‐time machine. Samples not presented here showed “undetermined” results across all three laboratories

Sample ID	P‐35S target						T‐nos target					
	Laboratory 1		Laboratory 2		Laboratory 3		Laboratory 1		Laboratory 2		Laboratory 3	
	Repetition 1	Repetition 2	Repetition 1	Repetition 2	Repetition 1	Repetition 2	Repetition 1	Repetition 2	Repetition 1	Repetition 2	Repetition 1	Repetition 2
Sample 2	Undetermined	Undetermined	Undetermined	Undetermined	Undetermined	Undetermined	Undetermined	Undetermined	42.4188	Undetermined	Undetermined	Undetermined
Sample 3	Undetermined	Undetermined	Undetermined	43.4346	Undetermined	Undetermined	Undetermined	Undetermined	Undetermined	46.0056	Undetermined	Undetermined
Sample 8	Undetermined	Undetermined	Undetermined	Undetermined	Undetermined	Undetermined	Undetermined	Undetermined	45.0097	Undetermined	Undetermined	Undetermined
Sample 9	Undetermined	Undetermined	Undetermined	Undetermined	Undetermined	Undetermined	Undetermined	Undetermined	Undetermined	44.2134	Undetermined	Undetermined
Sample 10	Undetermined	42.6236	Undetermined	Undetermined	Undetermined	Undetermined	Undetermined	Undetermined	Undetermined	Undetermined	Undetermined	Undetermined
Sample 12	Undetermined	Undetermined	Undetermined	Undetermined	Undetermined	39.7797	Undetermined	Undetermined	Undetermined	Undetermined	Undetermined	Undetermined
Sample 13	Undetermined	Undetermined	Undetermined	Undetermined	Undetermined	Undetermined	Undetermined	Undetermined	42.3097	41.9377	Undetermined	Undetermined
Sample 15	45.1385	43.1390	39.5828	39.2884	37.7255	37.8464	Undetermined	Undetermined	41.2294	41.4798	Undetermined	Undetermined
Sample 23	Undetermined	42.3278	Undetermined	38.9508	40.0059	36.7199	Undetermined	Undetermined	Undetermined	Undetermined	Undetermined	Undetermined
Sample 25	Undetermined	Undetermined	Undetermined	Undetermined	Undetermined	Undetermined	Undetermined	Undetermined	Undetermined	42.2851	Undetermined	Undetermined
Sample 26	Undetermined	Undetermined	40.8831	Undetermined	Undetermined	Undetermined	Undetermined	Undetermined	Undetermined	Undetermined	Undetermined	Undetermined
Sample 27	Undetermined	Undetermined	Undetermined	Undetermined	Undetermined	Undetermined	Undetermined	Undetermined	42.3586	43.6987	46.9756	45.9227
Sample 29	Undetermined	Undetermined	Undetermined	39.1318	38.7853	Undetermined	Undetermined	Undetermined	Undetermined	Undetermined	Undetermined	Undetermined
Sample 30	45.9439	Undetermined	Undetermined	Undetermined	Undetermined	Undetermined	Undetermined	Undetermined	Undetermined	Undetermined	Undetermined	Undetermined
Sample 32	46.0936	46.6986	Undetermined	Undetermined	Undetermined	Undetermined	Undetermined	Undetermined	46.8014	Undetermined	Undetermined	Undetermined
Sample 42	Undetermined	Undetermined	39.6490	Undetermined	Undetermined	Undetermined	Undetermined	Undetermined	Undetermined	Undetermined	Undetermined	Undetermined
Sample 45	Undetermined	43.8125	Undetermined	39.4448	Undetermined	Undetermined	Undetermined	Undetermined	Undetermined	Undetermined	Undetermined	Undetermined
Sample 47	Undetermined	Undetermined	Undetermined	46.5786	Undetermined	Undetermined	Undetermined	Undetermined	Undetermined	Undetermined	Undetermined	Undetermined
Sample 55	Undetermined	Undetermined	Undetermined	Undetermined	Undetermined	Undetermined	Undetermined	Undetermined	Undetermined	43.2296	Undetermined	Undetermined

The samples followed the amplification pattern typical of a low‐level presence (LLP) sample, in which amplification occurs in the final cycles of quantification of the run program (Gerdes, Busch, & Pecoraro, 2014). The average Cq for the screening assay was 42.3542 of 50 cycles program.

Due to the inconsistent amplification results within and across laboratories, we decided to apply a statistical approach to determine transgene presence using frequency distribution. This approach was based on the previous study of Gerdes et al. (2014), which used frequency distribution to investigate the experimental measurement variability for the quantification of very LLP of transgenes. Each laboratory performed a ten replicate assay for those samples with at least one amplification result. Only the laboratory that got an original amplification result on a sample went on to run a ten replicates assay because each laboratory received and worked with a different subsample (see Table 3). Table 3 shows the number of times amplification signals were detected during these ten replicates assays. Normalized Cq values of each replicate in these assays are provided in supplementary file 1.

**Table 3:** Number of amplification runs for the ten replicates for each sample. The results for the two transgenic targets (P35S and TNOS) are presented in separate columns. The ten replicates were only performed by the laboratories that obtained amplification in the first screening runs. Samples not presented here showed “undetermined” results in all three laboratories

Sample ID	P‐35S target			T‐nos target		Result interpretation
	Laboratory 1	Laboratory 2	Laboratory 3	Laboratory 2	Laboratory 3	
Sample 2	—	—	—	3	—	Likely negative
Sample 3	—	2	—	2	—	Likely negative
Sample 8	—	—	—	0	—	Likely negative
Sample 9	—	—	—	7	—	Likely positive
Sample 10	6	—	—	—	—	Likely positive
Sample 12	—	—	0	—	—	Likely negative
Sample 13	—	—	—	10	—	Likely positive
Sample 15	9	5	8	7	—	Likely positive
Sample 23	4	2	5	—	—	Likely positive
Sample 25	—	—	—	1	—	Likely negative
Sample 26	—	2	—	—	—	Likely negative
Sample 27	—	—	—	—	3	Likely negative
Sample 29	—	0	1	—	—	Likely negative
Sample 30	1	—	—	—	—	Likely negative
Sample 32	8	—	—	7	—	Likely positive
Sample 42	—	0	—	—	—	Likely negative
Sample 45	0	0	—	—	—	Likely negative
Sample 47	—	1	—	—	—	Likely negative
Sample 55	—	—	—	1	—	Likely negative

The frequency distribution of amplification results among the ten replicates performed on the samples in question confirmed six of the 19 samples that originally presented amplification signals in the previous screening assay. In such cases, samples were interpreted as “likely positives” because 50% or more of the PCR runs presented amplification signals for one of the transgenic targets. Samples with less than five Cq values for the ten replicates assay were scored “likely negative.” As no specific guidelines are available on how to interpret inconsistent amplification results derived from LLP samples, we chose the cut‐off value of 50%. We articulate on why we made this choice further in the discussion section below.

**Figure 3:**
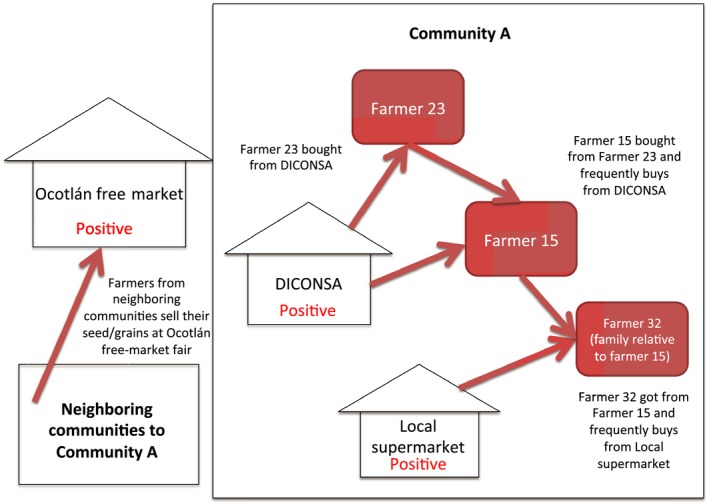
Graphical representation of seed management and sharing network connected to the positive samples found in this study

The ten replicates assay results revealed a total of six positive samples for either P‐35S or T‐nos transgenic elements, with sample 15 having amplification for both elements. A summary description of seed type and origin of the six positive samples is presented in Table 4. Interestingly, no positive results were obtained from samples collected at Community B. One of the samples (sample 9) was obtained from farmers from a neighboring community to Community A selling their seeds in the largest free market fair at the municipality of Ocotlán de Morelos. Samples 10 and 13 were obtained from shops at the city center of Community A, the local supermarket and DICONSA, respectively. The three remaining samples were from farmers, which are neighbors that engage in a seed sharing network and in which (at least one) reported having previously purchased and planted grain from local stores (see Fig. 3).

**Table 4:** Data description of positive samples

Sample ID	Seed type	Seed origin	Community
Sample 9	Yellow landrace variety	Free market (fair) at Ocotlán de Morelos municipality	Community A
Sample 10	White maize (unknown if an open pollinated variety or hybrid; *maiz blanco redondo* in Spanish).	Local supermarket	Community A
Sample 13	White maize (unknown if an open pollinated variety or hybrid)	DICONSA	Community A
Sample 15	White maize (unknown if an open pollinated variety or hybrid)	Farmer 15	Community A
Sample 23	White maize (unknown if an open pollinated variety or hybrid)	Farmer 23	Community A
Sample 32	White maize (unknown if an open pollinated variety or hybrid)	Farmer 32	Community A

By the time we visited farmers in Community A, many of them had already sown their plots with their landrace seeds. Most of them still had some seeds and grains left for their own consumption but others did not. This was the case in Sample 23, in which the farmer gave us seeds that had been bought at DICONSA because they had run out of their own seeds/grains. According to this farmer, it is not usual that they sow DICONSA seeds but it has been done in the past. This elderly farmer was, however, revealed through the interview to be a resource of both grains and seeds for other farmers in the community.

Indeed, the farmer who gave us Sample 15 confirmed that she had bought these seeds from Farmer 23 because she had also run out of her own seeds that year. Farmer 15 also confirmed that she grows seeds bought from other farmers or shops within the community when her seeds are not enough for the next year, similar to the case of Sample 32. Farmer 32, who is a relative of Farmer 15, also bought seeds from Farmer 23 and frequently buys seeds from the local stores (e.g., Supermarket) for food and growing purposes. It is clear from the interviews that the farmers whose seeds tested positive engage in a seed sharing network and had delivered grains that originally came from the DICONSA store but which are now being grown by some of the farmers within Community A. In addition, the other seed lots that tested positive were from local stores and markets (Samples 9 and 10) that are also sources of seed for many farmers in that community.

## 4 DISCUSSION

### 4.1 Are there transgenes in Mexican landrace maize?

The potential for transgene flow into landraces and wild relatives is a well‐recognized biosafety issue around the world and therefore an important component of the regulatory risk assessment performed on GM crops prior to their approval for cultivation. The case of transgenes in traditional maize landrace varieties, first reported in Mexico 15 years ago (Quist & Chapela, 2001) has drawn attention to the real possibility of contaminating crop varieties at their center of origin and diversity. However, the presence of transgenes in this case has remained under debate as the studies published afterward have shown contradictory results. Each of the previous studies performed on this topic used a different method and therefore a conclusive opinion about the presence of transgenes in Mexican landrace maize has been debated. Our study revisited the case of transgene flow into Mexican maize and used a novel approach based on socio‐biological analysis of contrasting farmer communities and seed management systems to investigate how different social and biological factors may affect the results of transgene detection and impact the spread of transgenes in Mexico.

Our study confirmed that socio‐biological factors, such as seed saving and sharing practices, communitarian organization, and land tenure arrangements, are important determinants affecting the frequency of transgene presence and the potential for spread within a community. This means that such social practices and arrangements may also be used as a resource to minimize the potential for or scale of transgene flow. As expected, our results show that communities in which seed sharing practices include the cultivation of seeds and/or grains from unknown varieties, such as DICONSA grains, are more vulnerable to transgene spread into their landrace varieties, which are usually cultivated in parallel. Even though farmers predominantly rely on local seed sources in Community A, infrequent cultivation of unknown seed or grains was demonstrated as serving as a source of transgene flow into the community—transgenes being detected in 20% of the samples collected from farmers in this community and the seed and grain markets they use. In contrast, we did not detect any transgenes in the samples from Community B located at Sierra Mixe in the Istmo region. These farmers are geographically isolated from other maize growing areas and their seed sharing practices and communitarian organization specifically avoid external sources of maize seeds and grains. In this case, transgenes were absent and unlikely to be present and spread to the same extent as in Community A.

Transgenes were already found absent and present at different frequencies in several regions across Mexico and also within the Oaxaca region, where our study was conducted (Dyer et al., 2009; Ortiz‐García et al., 2005a; Piñeyro‐Nelson et al., 2009a, 2009b; Quist & Chapela, 2001). Each of these studies used a different method to either sample maize, to conduct transgene detection analysis, and/or to interpret their results. Several critiques have also followed their publication (Christou, 2002; Cleveland et al., 2005; Kaplinsky et al., 2002; Metz & Fütterer, 2002; Schoel & Fagan, 2009). However, all these studies shared a common approach, sampling farmer seeds and fields without collecting information on the social context in which these seeds were produced and maintained over time. This is a kind of “population genetics approach,” in which the presence of a gene or transgene is the only aspect under analysis and very little can be inferred and estimated about the spread of transgenes into other communities and regions. Even when the same fields were sampled over years using the same method, researchers obtained different results for transgene frequency in these localities (Ortiz‐García et al., 2005a; Piñeyro‐Nelson et al., 2009a). Moreover, even when large sample sizes were used (about 9,000 leaf samples per community in Piñeyro‐Nelson et al., 2009a or 50,126 kernels analyzed in one growing season in Ortiz‐García et al., 2005a), they did not yield conclusive results either. This is because seed saving and sharing practices can vary each year, even for the same farmer. For example, if climatic conditions do not allow the crop to yield sufficient harvest, farmers are more likely to lack their own seeds for the next season and might then sow purchased or exchanged seeds.

Because we cannot test samples from every farmer in every Mexican community every year, it is crucial to develop research approaches that can start to provide a better understanding of seed management systems and practices in different communities and to use this knowledge to help anticipate where, how, and why transgene might be present in Mexican maize landraces as well as where spread is most likely to occur at a rapid rate. Some previous studies have indeed acknowledged that the model parameters used were based on survey data gathered specifically for a particular region of Mexico and that such parameters are likely to vary across different maize agricultural systems (Dyer et al., 2009; Piñeyro‐Nelson et al., 2009a). These authors pointed out that future studies should therefore analyze the effect of contrasting production and seed management conditions on transgene frequency distributions and their detection probabilities (Piñeyro‐Nelson et al., 2009a). In addition, the need to understand the interactions between formal and informal seed systems and grain markets in centers of crop origin and diversification has been highlighted (Dyer et al., 2009). Therefore, our study approach was a successful case study examining how different societal and biological factors can affect transgene frequency in a community and highlighted just how difficult it is to make generalizations in the case of maize in Mexico due to its large diversity. Furthermore, this study has also emphasized how difficult controlling the spread of transgenes will be for communities that both regularly share seeds within and outside the community in informal markets and purchase seeds from contaminated grain stores in the formal market. Our research has also revealed that the challenge of eliminating and/or controlling transgene spread may only be amplified by a lack of knowledge within the communities concerning GMOs. Identifying seed management practices that make communities particularly vulnerable to transgene flow (such as regular seed exchanges outside the community, purchasing and planting unknown seeds, planting materials sold as grain, mixing of hybrid and landrace varieties) may usefully help to identify areas where the likelihood of contamination (now or in the future) is relatively high and where information and education campaigns (and potentially also regular detection and monitoring work) would be beneficial if transgene flow is to be controlled.

Our results suggest that transgenes are highly likely to be present in Mexican maize landraces and importantly that the extent and frequency at which transgenes can be found will very much depend on the seed management practices and societal characteristics of the different communities engaged in maize farming. Therefore, future analysis of transgene presence in maize landraces in Mexico, as well as the development of potential management strategies for controlling its spread, must include research and work on socio‐cultural elements to better understand the role of seed management systems for how transgenes may enter and move through communities.

### 4.2 Difficulties in detecting transgenes in landraces and wild relatives

Studies reporting inconsistent results on transgenes in Mexican maize have created a focused dispute on what is the best/most appropriate and reliable method to use when seeking to detect transgenes in landraces and wild relatives. Although transgene detection methods and techniques have certainly evolved since the initial report of their presence in Mexico in 2001 (Quist & Chapela, 2001), there is still no scientific agreement or internationally agreed and standardized approach that would be specific for the unique challenges associated with detection of transgenes in landraces and wild relatives.

Our study used the most sensitive and robust method for transgene screening, as well as validated protocols for GMO analysis and DNA extraction. In addition, this study also applied an interlaboratorial analysis to confirm results. However, even when the same sample set and the same validated protocols were applied, we obtained inconsistent results on the presence of transgenic elements across the three laboratories and even across the technical replicates performed within the same laboratories. This is arguably linked to the fact that although there are a number of validated methods and protocols for transgene detection, none of them are specifically developed and fit for the purpose of detecting transgenes in landraces and wild relatives. For example, transgenic DNA sequences or transgenic proteins must be intact and/or expressing to be detectable by current methods and because landraces and wild relatives have highly heterogeneous genomes, these sequences might be subject to variation due to transposon activity or crossing over events (Quist & Chapela, 2001). In addition, methodological controls are typically based on endogenous genes or proteins and these might differ in landraces and wild relatives. Existing guidelines outlining the technical issues related to the estimation of measurement uncertainty (MU) do consider parameters associated with the dispersion of the values (Trapmann et al., 2009). However, once again these are relative to the MU associated with an analytical result only when deciding whether that result falls within the legislation for food and feed control purposes.

Detection methods also have intrinsic problems that become particularly relevant when working with landraces or wild relatives. Our results indicate two major problems in our sample set analysis: DNA isolation of heterogeneous samples and transgene presence at near LOD levels. Heterogeneous samples tend to produce different subsamples even when ground to a fine powder (Berben et al., 2008) and that certainly might explain why the three laboratories got different transgene presence results in our study. Because each of the three laboratories received a unique subsample of 1.5 g derived from a larger sample of 100 g, each may have received different concentrations of transgenic DNA sequences. This issue has been discussed in the critiques of publications reporting positive results in the Mexican case but it is important to note that although we may see divergence in results across different subsamples, this does not on its own negate the validity of positive findings.

The heterogeneous presence of transgenes in subsamples is also true for samples containing transgenes at near LOD concentrations. In previous studies, LOD levels were established by visual inspection of a dilution series in an agarose gel (Ortiz‐García et al., 2005a; Piñeyro‐Nelson et al., 2009a, 2009b; Quist & Chapela, 2001) and, therefore, it is not possible to verify whether this was also the case for the inconsistent results reported earlier. In fact, when transgene flow or introgression has taken place in a landrace or wild relative, the copy numbers of PCR targets (i.e., transgene elements) will most likely differ depending on both the number of transgene events that have occurred and the number of crossings. If two screening targets are present in a GMO but with different insert copy numbers, for example one and four, the relative LOD for these will differ fourfold for a DNA solution obtained solely from that GMO (Holst‐Jensen et al., 2012).

The only previous study reporting no detection of transgenes in communities of Oaxaca was criticized using the argument that the results obtained could have involved false negatives due to the expression of a variety of secondary metabolites in landrace samples inhibiting PCR amplification (Ortiz‐García et al., 2005a; Piñeyro‐Nelson et al., 2009a, 2009b). However, the detection service provider for that study analysis confirmed that they had tested for PCR inhibitors through inhibition tests and did not find any such molecules (Schoel & Fagan, 2009). In addition, recombination in homozygous or hemizygous (at least two times less) transgenic plants can occur (Molinier, Ries, Bonhoeffer, & Hohna, 2004). Therefore, some events can take place in the target DNA sequences that might avoid or make difficult primer annealing. Uncertainty does, however, remain regarding how much interference these characteristics might create for PCR efficiency in detecting transgenes and internal control genes and how to adapt current methodologies to overcome such challenges.

It is therefore important that the limitations of existing approaches to detecting transgenes in landraces and wild relatives are recognized, both within the scientific community but also within national and international policy contexts. Revisiting the iconic case of transgene flow into landraces of maize in Mexico 15 years ago has revealed that future work in this field would benefit from more socio‐biological approaches that would include gathering information on the social context in which environmental samples are taken. Furthermore, better guidance on establishing the ability to detect low‐level traces of transgenes, on how to validate results (e.g., using both interlaboratory validation and multiple methods) as well as the minimum information required for reporting transgene detection results is necessary. The importance of conserving genetic biodiversity in crop plants in the face of the rapid expansion of new biotechnological organisms, especially across mega diverse countries, makes the establishment of good practices for transgene detection and monitoring even more urgent and pressing now than it was 15 years ago.

## CONFLICT OF INTEREST

None declared.

## AUTHOR CONTRIBUTIONS

SZA, FRL, and FW planned the fieldwork. SZA, RON, NM, GAS, and MT planned the laboratory analysis. SZA and FRL conducted the fieldwork. SZA, NM, GAS, and MT conducted the laboratory experiments. SZA, FRL, FW, NM, GAS, and MT participated in a workshop to discuss the laboratory results and further analysis. SZAT and FW wrote the draft manuscript text and all co‐authors revised and agreed with the manuscript final version.

## DATA ACCESSIBILITY

No large datasets requiring external archiving were used or generated during this study. All data related to the sampling locations, methods, and results are provided in the main body of the paper.

## Endnote

1 Locations are approximate so as to preserve the confidentiality of the participants in the study and to avoid any potential harm that the results of the research may carry for any community member or authority involved.
