# Supplementary material for: Transgene flow in Mexican maize revisited: Socio‐biological analysis across two contrasting farmer communities and seed management systems
Source: Ecol Evol. 2017 Oct 11;7(22):9461–72. doi: 10.1002/ece3.3415 (PMC5696427; doi:10.1002/ece3.3415)
Supplement: Supplementary file 1 [file ECE3-7-9461-s001.pdf]

Transgene flow in Mexican maize revisited: socio-biological analysis across two contrasting communities and seed management systems

Sarah Z. Agapito-Tenfen[1], Flor R. Lopez[1], Narmeen Mallah[2], Gretta Abou-Slemayne[2], Miluse Tritkova[3], Angelika Hilbeck[3], Rubens O. Nodari[4] and Fern Wickson[1]*

Table 2. Cycle of quantification (Cq) for tenplicates for each sample. The two gene targets (P35S and TNOS) results are presented in separate columns. Tenplicates were only performed by the laboratories that obtained amplification in the first screening runs. 'Udetermined' results were obtained when no fluorescent signal was detected by the real-time machine. Samples not presented here showed 'undetermined' results in all three laboratories.

Sample ID	P-35S target			T-nos target		Results interpretation
	Laboratory 1	Laboratory 2	Laboratory 3	Laboratory 2	Laboratory 3	
Sample 2				43.8939		
Sample 2				Undetermined		
Sample 2				Undetermined		
Sample 2				38.7119		
Sample 2				Undetermined		
Sample 2				Undetermined		Likely negative
Sample 2				Undetermined		
Sample 2				45.1731		
Sample 2				Undetermined		
Sample 2				Undetermined		
Sample 3		Undetermined		Undetermined		
Sample 3		39.9048		Undetermined		
Sample 3		Undetermined		49.2268		
Sample 3		Undetermined		Undetermined		
Sample 3		Undetermined		46.3161		Likely negative

Sample 3		Undetermined	Undetermined	Likely negative
Sample 3		Undetermined	Undetermined	
Sample 3		40.9677	Undetermined	
Sample 3		Undetermined	Undetermined	
Sample 3		Undetermined	Undetermined	
Sample 8			Undetermined	
Sample 8			Undetermined	
Sample 8			Undetermined	
Sample 8			Undetermined	
Sample 8			Undetermined	Likely negative
Sample 8			Undetermined	
Sample 8			Undetermined	
Sample 8			Undetermined	
Sample 8			Undetermined	
Sample 8			Undetermined	
Sample 9			45.5834	
Sample 9			Undetermined	
Sample 9			44.9332	
Sample 9			Undetermined	
Sample 9			46.9922	Likely positive
Sample 9			45.3400	
Sample 9			Undetermined	
Sample 9			36.3834	
Sample 9			Undetermined	
Sample 9			Undetermined	
Sample 10	Undetermined			
Sample 10	42.7492			
Sample 10	42.3411			
Sample 10	42.3943			

Sample 10	42.1256				Likely positive
Sample 10	Undetermined				
Sample 10	Undetermined				
Sample 10	49.0363				
Sample 10	Undetermined				
Sample 10	43.0241				
Sample 12			Undetermined		
Sample 12			Undetermined		
Sample 12			Undetermined		
Sample 12			Undetermined		
Sample 12			Undetermined		Likely negative
Sample 12			Undetermined		
Sample 12			Undetermined		
Sample 12			Undetermined		
Sample 12			Undetermined		
Sample 12			Undetermined		
Sample 13				41.9487	
Sample 13				42.4424	
Sample 13				41.1687	
Sample 13				42.1449	
Sample 13				43.2548	Likely positive
Sample 13				42.3512	
Sample 13				43.0073	
Sample 13				41.6195	
Sample 13				41.6833	
Sample 13				40.8850	
Sample 15	42.2658	Undetermined	38.7757	43.6966	
Sample 15	41.3420	38.3857	38.3893	35.6413	
Sample 15	40.9599	Undetermined	40.7197	41.6961	

Sample 15	40.3920	Undetermined	36.2072	44.1500	
Sample 15	40.9655	Undetermined	Undetermined	Undetermined	Likely positive
Sample 15	40.8670	41.0659	39.4073	44.8792	
Sample 15	Undetermined	Undetermined	39.9811	Undetermined	
Sample 15	40.1111	40.9322	Undetermined	42.1131	
Sample 15	40.6650	40.3591	40.6511	Undetermined	
Sample 15	41.1683	40.0375	37.7076	43.3320	
Sample 23	Undetermined	Undetermined	39.5876		
Sample 23	42.5974	43.2257	Undetermined		
Sample 23	Undetermined	40.4156	39.3056		
Sample 23	Undetermined	Undetermined	Undetermined		
Sample 23	Undetermined	Undetermined	Undetermined		Likely positive
Sample 23	42.9792	Undetermined	39.5331		
Sample 23	44.1078	Undetermined	Undetermined		
Sample 23	41.2226	Undetermined	Undetermined		
Sample 23	Undetermined	Undetermined	39.1737		
Sample 23	Undetermined	Undetermined	37.9411		
Sample 25				Undetermined	
Sample 25				43.0099	
Sample 25				Undetermined	
Sample 25				Undetermined	
Sample 25				Undetermined	Likely negative
Sample 25				Undetermined	
Sample 25				Undetermined	
Sample 25				Undetermined	
Sample 25				Undetermined	
Sample 25				Undetermined	
Sample 26		Undetermined			
Sample 26		Undetermined			

Sample 26		48.5509			
Sample 26		42.3256			
Sample 26		Undetermined			Likely negative
Sample 26		Undetermined			
Sample 26		Undetermined			
Sample 26		Undetermined			
Sample 26		Undetermined			
Sample 26		Undetermined			
Sample 27				43.9532	
Sample 27				Undetermined	
Sample 27				Undetermined	
Sample 27				47.0159	
Sample 27				Undetermined	Likely negative
Sample 27				Undetermined	
Sample 27				30.8795	
Sample 27				Undetermined	
Sample 27				Undetermined	
Sample 27				Undetermined	
Sample 29		Undetermined	Undetermined		
Sample 29		Undetermined	Undetermined		
Sample 29		Undetermined	Undetermined		
Sample 29		Undetermined	39.9096		
Sample 29		Undetermined	Undetermined		Likely negative
Sample 29		Undetermined	Undetermined		
Sample 29		Undetermined	Undetermined		
Sample 29		Undetermined	Undetermined		
Sample 29		Undetermined	Undetermined		
Sample 29		Undetermined	Undetermined		
Sample 30	Undetermined				

Sample 30	Undetermined			
Sample 30	Undetermined			
Sample 30	Undetermined			
Sample 30	41.4594			
Sample 30	Undetermined			Likely negative
Sample 30	Undetermined			
Sample 30	Undetermined			
Sample 30	Undetermined			
Sample 30	Undetermined			
Sample 32	Undetermined		45.1723	
Sample 32	42.3166		46.0738	
Sample 32	41.8123		46.2573	
Sample 32	Undetermined		46.4554	
Sample 32	42.8576		46.9247	Likely positive
Sample 32	40.0453		45.2411	
Sample 32	42.5698		47.2882	
Sample 32	41.2622		Undetermined	
Sample 32	40.9051		Undetermined	
Sample 32	40.0920		Undetermined	
Sample 42		Undetermined		
Sample 42		Undetermined		
Sample 42		Undetermined		
Sample 42		Undetermined		
Sample 42		Undetermined		Likely negative
Sample 42		Undetermined		
Sample 42		Undetermined		
Sample 42		Undetermined		
Sample 42		Undetermined		
Sample 42		Undetermined		

Sample 45	Undetermined	Undetermined		
Sample 45	Undetermined	Undetermined		
Sample 45	Undetermined	Undetermined		
Sample 45	Undetermined	Undetermined		
Sample 45	Undetermined	Undetermined		Likely negative
Sample 45	Undetermined	Undetermined		
Sample 45	Undetermined	Undetermined		
Sample 45	Undetermined	Undetermined		
Sample 45	Undetermined	Undetermined		
Sample 45	Undetermined	Undetermined		
Sample 47		Undetermined		
Sample 47		42.2901		
Sample 47		Undetermined		
Sample 47		Undetermined		
Sample 47		Undetermined		Likely negative
Sample 47		Undetermined		
Sample 47		Undetermined		
Sample 47		Undetermined		
Sample 47		Undetermined		
Sample 47		Undetermined		
Sample 55			Undetermined	
Sample 55			Undetermined	
Sample 55			Undetermined	
Sample 55			Undetermined	
Sample 55			Undetermined	Likely negative
Sample 55			Undetermined	
Sample 55			Undetermined	
Sample 55			Undetermined	
Sample 55			49.5846	

Sample 55 Undetermined
